# Analysis of light-wave nonstaticity in the coherent state

**DOI:** 10.1038/s41598-021-03047-8

**Published:** 2021-12-14

**Authors:** Jeong Ryeol Choi

**Affiliations:** Department of Nanoengineering, Kyonggi University, Suwon, Gyeonggi-do 16227 Republic of Korea

**Keywords:** Mathematics and computing, Optics and photonics, Physics

## Abstract

The characteristics of nonstatic quantum light waves in the coherent state in a static environment is investigated. It is shown that the shape of the wave varies periodically as a manifestation of its peculiar properties of nonstaticity like the case of the Fock-state analysis for a nonstatic wave. A belly occurs in the graphic of wave evolution whenever the wave is maximally displaced in the quadrature space, whereas a node takes place every time the wave passes the equilibrium point during its oscillation. In this way, a belly and a node appear in turn successively. Whereas this change of wave profile is accompanied by the periodic variation of electric and magnetic energies, the total energy is conserved. The fluctuations of quadratures also vary in a regular manner according to the wave transformation in time. While the resultant time-varying uncertainty product is always larger than (or, at least, equal to) its quantum-mechanically allowed minimal value ($\hbar /2$), it is smallest whenever the wave constitutes a belly or a node. The mechanism underlying the abnormal features of nonstatic light waves demonstrated here can be interpreted by the rotation of the squeezed-shape contour of the Wigner distribution function in phase space.

## Introduction

A light wave in media can vary spatiotemporally according not only to the variation of electromagnetic parameters but to its interaction with matters as well. This may lead the wave being nonstatic^1–8^. Regarding this, the interaction of laser light with time-varying media has been a subject of great interest from the early days of modern physics^6–12^. Electromagnetic waves can be amplified or dissipated through the coupling of them, for example, with a plasma wave^6–10^. This outcome is applicable to several physical branches, such as the frequency shifts of light waves^8,9^, laser-driven wakefield accelerators^7,12^, plasma parametric amplification^4,13^, and harmonic generation^14^. A notable field among them is a production of terahertz/millimeter-waves via frequency shifts. The resources of other means for producing such waves are actually rare and limited^5^. It is also noteworthy that femtosecond light pulses produced by high power lasers are necessary in realizing ultrafast optical technology such as ion acceleration at multi-MeV energies^15,16^. Beside these, there are many other scientific and technological branches where the nonstatic waves are utilizable^17–20^.

From our recent report associated with light-wave nonstaticity^21^, it was known that nonstatic waves also take place even when the environment is static. Regarding this, the time behavior of nonstatic waves in the Fock states was analyzed fundamentally. Nonstatic waves in such a case show a peculiar property that the waves undergo collapse and expansion in turn periodically in quadrature space. The concept of the measure of nonstaticity has been introduced as a tool for estimating the magnitude of nonstatic character for such a wave^21^. In order for efficient manipulation and control of nonstatic waves, it is necessary to understand the mechanism how nonstatic light waves evolve.

The research for wave nonstaticity in a coherent state, as well as in the Fock states, may also deserve our attention. Coherent states are fundamental in quantum optics because they allow classical-like description of light waves. Glauber-type coherent states^22^ provide an elegant representation of wave evolution with Gaussianity. A paradigmatic research with coherent states is demonstrating quantum-classical correspondence which addresses how quantum behavior of a system develops to classicality^23^. It is well known that coherent-state description of a quantum state can also be extended to a wide range of branches in physics beyond quantum optics, such as atomic physics^24,25^, nuclear physics^26,27^, solid state mechanics^28,29^, biological systems^30,31^, etc.

Stimulated by the above consequence and associated research trends, we will investigate nonstatic waves in this work, focusing on the coherent state in a static environment. A quadratic invariant operator which follows the Liouville-von Neumann equation will be adopted for this purpose. Lots of dynamical properties of non-ideal physical systems including nonstatic light waves can be treated by means of such a dynamical invariant^32–34^. The reason why the invariant operator method is useful in this context is that a generalized quantum wave function of a light wave is obtained by utilizing an invariant operator instead of the direct use of the Hamiltonian.

How the wave nonstaticity affects the time evolutions of diverse physical quantities will be analyzed rigorously. The similarities and differences between the evolutions of nonstatic and static coherent waves will be clarified. Moreover, we will find a global profile of wave nonstaticity extended to the coherent state and provide graphics of the wave evolutions which display apparent nonstaticity. The phenomena of wave collapse and expansion in the coherent state will be illustrated and compared with those occurred in the Fock states. The behavior of quantum energy and quadrature fluctuations which accompany nonstatic coherent waves will also be addressed, examining their pattern/regularity in the evolution. Finally, we will try to elucidate the mechanism related to resultant wave-nonstaticity on the basis of the Wigner distribution function.

## Results and discussion

### Basic formulation and the invariant operator

We consider a light wave propagating through a non-conductive medium in which electric permittivity $\epsilon$ and magnetic permeability $\mu$ do not vary over time. Because the electromagnetic parameters are independent of time in this case, the medium is static to the wave packets evolving in it. The Hamiltonian for the light waves in such a static medium is given by $\hat{H}={\hat{p}^2}/{(2\epsilon )} + \epsilon \omega ^2 \hat{q}^2/2 ,$ where $\omega$ is the angular frequency of the form $\omega =k c$ whereas *k* is the wave number. Then, the wave velocity is constant and it is given by $c=1/\sqrt{\epsilon \mu }$.

By the way, as mentioned in the introduction part, nonstatic quantum waves can emerge in the Fock states in this static situation. Wave nonstaticity may also appear in other states in general, such as the coherent state, the squeezed state, and the thermal state. We investigate quantum wave phenomena associated with the nonstatic coherent state built up under the static circumstance. This will be carried out based on the complete analytical description of them.

To treat the light in a general way, let us see the invariant operator theory^32^. In fact, the Hamiltonian itself is an invariant operator for this system, because the Hamiltonian is a time-*in*dependent form that corresponds to a conserved energy. However, in order to treat the system more generally, we need to obtain a general form of an invariant $\hat{I}$ from the Liouville-von Neumann equation:

1 $$\begin{aligned} {d \hat{I}}/{d t} = {\partial \hat{I}}/{\partial t} + [\hat{I},\hat{H}]/(i\hbar ) = 0. \end{aligned}$$

By inserting the Hamiltonian into this equation, we derive a quadratic invariant operator as

2 $$\begin{aligned} \hat{I}= \hbar \omega \left[ \frac{\epsilon \omega }{2\hbar f(t)} \hat{q}^2 +\frac{f(t)}{2\epsilon \omega \hbar } \Bigg ( \hat{p}-\frac{\epsilon \dot{f}(t)}{2f(t)}\hat{q}\Bigg )^2 \right] , \end{aligned}$$

where *f*(*t*) is a time function that yields the nonlinear equation^21,33^

3 $$\begin{aligned} \ddot{f}(t) - \frac{[\dot{f}(t)]^2}{2f(t)} + 2\omega ^2 \left( f(t)- \frac{1}{f(t)}\right) =0. \end{aligned}$$

Although the time derivative of Eq. (2) results in zero, the invariant operator $\hat{I}$ is given in terms of *f*(*t*) that is related to the time evolution of the system. We consider a general solution for *f*(*t*), which is given by^21^:

4 $$\begin{aligned} f(t) = c_1 \sin ^2 \tilde{\varphi }(t)+ c_2 \cos ^2 \tilde{\varphi }(t) + c_3 \sin [2\tilde{\varphi }(t)], \end{aligned}$$

where $\tilde{\varphi }(t)=\omega (t-t_0) +\varphi$, $t_0$ is an initial time and $\varphi$ is a phase, whereas $c_1$, $c_2$, and $c_3$ are real constants that follow the condition

5 $$\begin{aligned} c_1c_2-c_3^2 = 1,~~~~~~c_1c_2 \ge 1. \end{aligned}$$

Without loss of generality, we restrict $\varphi$ within the range $-\pi /2 \le \varphi < \pi /2$ for convenience; the consideration of this range is enough because the period of Eq. (4) is $\pi$.

At this stage, we introduce an annihilation operator associated with the invariant, Eq. (2), which is of the form^33^

6 $$\begin{aligned} \hat{A}= \sqrt{\frac{\epsilon \omega }{2\hbar f(t)}}\left( 1- i \frac{\dot{f}(t)}{2\omega }\right) \hat{q} + i\sqrt{\frac{ f(t)}{2\epsilon \omega \hbar }} \hat{p}. \end{aligned}$$

Then, its Hermitian adjoint $\hat{A}^\dagger$ is a creation operator. These operators obey the boson commutation relation $[\hat{A},\hat{A}^\dagger ]=1$. Notice that the invariant operator can be rewritten in terms of these generalized ladder operators to be

7 $$\begin{aligned} \hat{I}= \hbar \omega \bigg ( \hat{A}^\dagger \hat{A} +\frac{1}{2}\bigg ). \end{aligned}$$

If we denote the eigenfunctions of $\hat{I}$ as $\langle q |\Phi _n(t) \rangle$ ($n=0,1,2,\ldots$), we can obtain them from the eigenvalue equation $\hat{I}\langle q |\Phi _n(t) \rangle = \lambda _n \langle q |\Phi _n(t) \rangle$, while their formula is provided in Eq. (46) in “Methods” section (the last section). Here, the eigenvalues are given by $\lambda _n = \hbar \omega ( n +{1}/{2})$. Notice that the wave functions in the Fock states are represented by $\langle q |\Phi _n(t) \rangle$ as shown in Eq. (45). For the basic of solving the eigenvalue equation of an invariant operator, refer to Refs.^32,35^.

From inverse representations of Eq. (6) together with its conjugate equation ($\hat{A}^\dagger$), we can readily have the formula of $\hat{q}$ and $\hat{p}$, such that

8 $$\begin{aligned} \hat{q}= & {} \sqrt{\frac{\hbar f(t)}{2\epsilon \omega }} (\hat{A} + \hat{A}^\dagger ), \end{aligned}$$

9 $$\begin{aligned} \hat{p}= & {} -i \sqrt{\frac{\hbar \epsilon \omega }{2f(t)}} \left[ \left( 1+i \frac{\dot{f}(t)}{2\omega }\right) \hat{A} -\left( 1-i \frac{\dot{f}(t)}{2\omega }\right) \hat{A}^\dagger \right] . \end{aligned}$$

These formulae are useful when we investigate the behavior of quantum observables such as quadrature fluctuations and quantum energy.

As is well known, the Glauber coherent state is the eigenstate of an annihilation operator. A generalized wave function in the coherent state will be derived by evaluating the eigenstate of $\hat{A}$ given in Eq. (6) in the subsequent subsection. We will use this wave function as a basic tool for unfolding quantum theory of wave nonstaticity.

### Wave nonstaticity in the coherent state

To obtain the analytical description of the wave function in the coherent state, let us see the eigenstate of $\hat{A}$. If we write the eigenvalue equation of $\hat{A}$ in the form

10 $$\begin{aligned} \hat{A} |A \rangle =A |A \rangle , \end{aligned}$$

$|A \rangle$ is the coherent state. By solving Eq. (10) using Eq. (6) in the configuration space in a straightforward way, we have the coherent state as

11 $$\begin{aligned} \langle q |A \rangle = \root 4 \of {\frac{\zeta (t)}{\pi }} \exp \Bigg [ -\frac{\zeta (t)}{2} \bigg ( 1- i \frac{\dot{f}(t)}{2\omega } \bigg )q^2 + \sqrt{{2\zeta (t)}} A q -\frac{1}{2} |A|^2-\frac{1}{2} A^2 \Bigg ], \end{aligned}$$

where $\zeta (t) = {\epsilon \omega }/{[\hbar f(t)]}$. From this wave function, we can investigate various properties of the nonstatic light wave in the coherent state.

If $c_1=c_2=1$ and $c_3=0$, the wave undergoes no nonstaticity. The wave nonstaticity occurs only when $c_1$ and/or $c_2$ deviate from unity. If such deviations are large, the wave becomes highly nonstatic. Regarding this, the measure of nonstaticity associated with Eq. (11) is the same as that in the Fock states, which is of the form^21^

12 $$\begin{aligned} D_\mathrm{} = \frac{\sqrt{(c_1+c_2)^2-4}}{2\sqrt{2}}. \end{aligned}$$

We now see the eigenvalue *A* of Eq. (10) in detail. For this purpose, let us denote the solutions of the classical equations of motion (second-order differential equations) for canonical variables *q* and *p* as $Q_\mathrm{cl}(t)$ and $P_\mathrm{cl}(t)$, respectively. Then, the eigenvalue is given in terms of them as

13 $$\begin{aligned} A= \sqrt{\frac{\epsilon \omega }{2\hbar f(t)}}\left( 1- i \frac{\dot{f}(t)}{2\omega }\right) Q_\mathrm{cl}(t) + i\sqrt{\frac{ f(t)}{2\epsilon \omega \hbar }} P_\mathrm{cl}(t). \end{aligned}$$

From fundamental mechanics, we can represent

14 $$\begin{aligned} Q_\mathrm{cl}(t)= & {} Q_0 \cos \tilde{\theta }(t), \end{aligned}$$

15 $$\begin{aligned} P_\mathrm{cl}(t)= & {} \epsilon \frac{d Q_\mathrm{cl}(t)}{dt}, \end{aligned}$$

where $\tilde{\theta }(t)=\omega (t-t_0) +\theta _0$, $\theta _0$ is an arbitrary phase at $t_0$. The eigenvalue *A* can be rewritten in terms of an amplitude and a phase, such that

16 $$\begin{aligned} A=A_0 e^{i\kappa }, \end{aligned}$$

where

17 $$\begin{aligned} A_0= & {} \Bigg \{ \frac{\epsilon \omega }{2\hbar }\Bigg [\frac{\cos ^2 \tilde{\theta }(t)}{f(t)}+ \Bigg ( \frac{\dot{f}(t)}{2\omega \sqrt{f(t)}}\cos \tilde{\theta }(t) +\sqrt{f(t)}\sin \tilde{\theta }(t) \Bigg )^2 \Bigg ] \Bigg \}^{1/2}Q_0 , \end{aligned}$$

18 $$\begin{aligned} \kappa= & {} \tan ^{-1}\bigg [ -\bigg ( \frac{\dot{f}(t)}{2\omega } + f(t) \tan \tilde{\theta }(t) \bigg ) \bigg ]. \end{aligned}$$

We can easily check that the differentiation of $A_0$ with respect to time results in zero. This means that $A_0$ is a time-constant. Note that $A_0$ can also be expressed in a simple form as

19 $$\begin{aligned} A_0 = \sqrt{\frac{\epsilon \omega }{2\hbar }f(t_1)} Q_0, \end{aligned}$$

where $t_1$ is a particular time that is given by $t_1 = t_0 + (\pi /2-\theta _0)/\omega$. From a direct differentiation of Eq. (18) with time, we have

20 $$\begin{aligned} \frac{d\kappa }{dt} = - \frac{\omega }{f(t)}. \end{aligned}$$

Hence, we can put $\kappa$ in the form

21 $$\begin{aligned} \kappa = -\bigg (\omega \int _{t_0}^t f^{-1}(t') dt' + \theta \bigg ), \end{aligned}$$

where $\theta$ is a phase. Thus, we conclude that

22 $$\begin{aligned} A(t) = A(t_0) e^{-i\omega T(t)}, \end{aligned}$$

where $$A(t_0) = A_0 e^{-i\theta }$$ and $$T(t) = \int _{t_0}^t f^{-1}(t')dt'$$.

The integration in *T*(*t*) can be performed and this leads to^21,36^

23 $$\begin{aligned} T(t) = G(t) - G(t_0) +{\mathcal G}(t)/\omega , \end{aligned}$$

where

24 $$\begin{aligned} G(\tau ) = \frac{1}{\omega }\tan ^{-1} \{ c_3+c_1\tan [\omega (\tau -t_0) +\varphi ] \}, \end{aligned}$$

while ${\mathcal G}(t)=\pi \sum _{m=0}^{\infty }u[t-t_0-(2m+1)\pi /(2\omega )+\varphi /\omega ]$ and *u*[*x*] is the Heaviside step function. By inserting Eq. (23) with Eq. (24) into Eq. (21), we can have the formula of $\kappa$. Notice that $\kappa$ obtained in such a way is continuous over time because the discontinuity originated from the characteristic of the arctangent function is compensated by introducing the step function.

On the other hand, the former expression of $\kappa$ given in Eq. (18) is discontinuous over time, although its time derivative gives the same result as that of the new $\kappa$ which was mentioned a little while ago. For this reason, we will use Eq. (21) with Eqs. (23) and (24) as the expression of $\kappa$ in the subsequent analysis of the nonstatic coherent wave.

**Figure 1:**
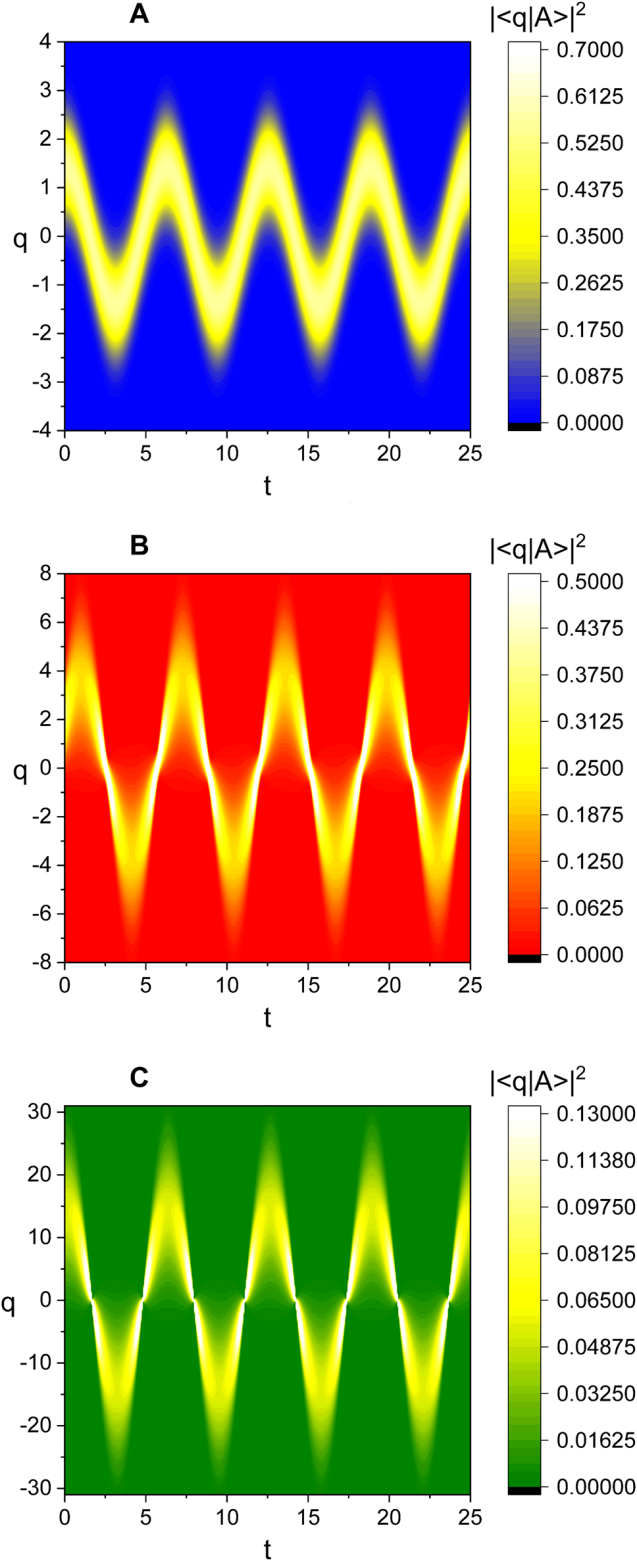
Time evolution of the probability density $|\langle q |A \rangle |^2$ where ($c_1$, $c_2$) are (1, 1) for A, (5, 2) for B, and (1, 100) for C. The measure of nonstaticity is 0.00 for A, 2.37 for B, and 35.70 for C. We have used $\omega =1$, $A_0=1$, $\hbar =1$, $\epsilon =1$, $t_0=0$, and $\varphi =\theta =0$. We see from Eq. (5) that the allowed values of $c_3$, when $c_1$ and $c_2$ have been determined, are two: One is positive and the other is negative. Among them, we choose a positive one as the value of $c_3$ in this and all subsequent figures for convenience.

Because we know the complete formula of *A*(*t*) from Eq. (22) and subsequent equations at this stage, it is possible to investigate the characteristics of the wave function given in Eq. (11). Figure 1 is the time evolution of the probability density $|\langle q |A \rangle |^2$ associated with that wave function. The formula of *A*(*t*) given in Eq. (22) will also be used subsequently in order to investigate other physical quantities in the coherent state. By the way, if we take $c_3 =0$ and a different formula of *A* similar to Eq. (13) instead of the one given in Eq. (22), the coherent state developed here reduces to that of Ref.^33^.

*p*-space analysis of the coherent state may also be necessary for the complete understanding of the evolution of the nonstatic wave. *p*-space eigenfunction of the invariant operator, i.e., the wave function in *p*-space can be obtained from the Fourier transformation:

25 $$\begin{aligned} \langle {p}|A \rangle = \frac{1}{\sqrt{2\pi \hbar }} \int _{-\infty }^{\infty } \langle q|A \rangle e^{-i {p}{q}/\hbar } d q. \end{aligned}$$

The exact formula of $\langle {p}|A \rangle$ is represented in “Methods” section.

**Figure 2:**
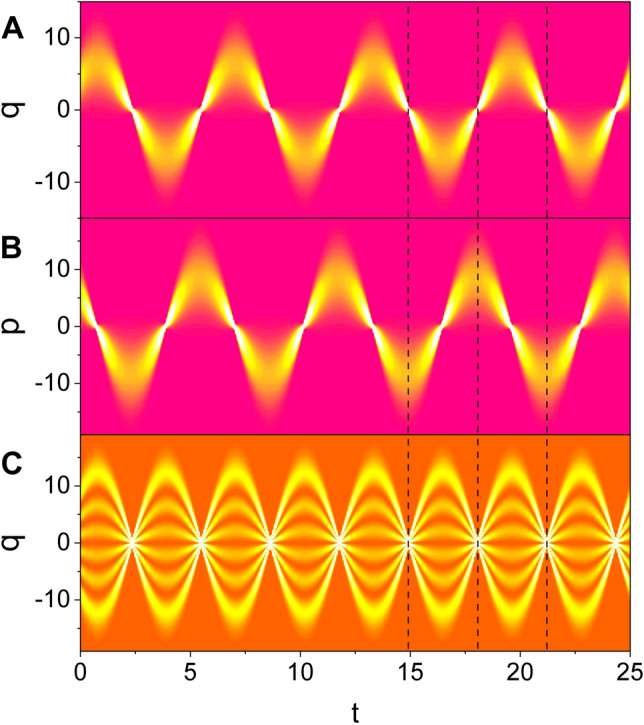
Comparison between time evolutions of different probability densities for nonstatic waves. (**A**) is $|\langle q |A \rangle |^2$, (**B**) is $|\langle p |A \rangle |^2$, and (**C**) is $|\langle q |\Psi _n \rangle |^2$ where $n=5$. We have used ($c_1$, $c_2$) = (10, 10), $\omega =1$, $A_0=1$, $\hbar =1$, $\epsilon =1$, $t_0=0$, and $\varphi =\theta =0$.

The three kinds of the probability densities, $|\langle {q}|A \rangle |^2$, $|\langle {p}|A \rangle |^2$, and $|\langle {q}|\Psi _n \rangle |^2$ ($n=1,2,3,\cdots$), have been compared to each other in Fig. 2, where $\langle {q}|\Psi _n \rangle$ are Fock-state wave functions that were previously investigated in Ref.^21^. The formula of $\langle {q}|\Psi _n \rangle$ has been represented in “Methods” section for convenience. The phase difference between the evolutions of $|\langle {q}|A \rangle |^2$ and $|\langle {p}|A \rangle |^2$ is $\pi /2$. From a careful comparison of Fig. 2A with Fig. 2C, we confirm that $|\langle {q}|A \rangle |^2$ constitutes a node (a belly) whenever $|\langle {q}|\Psi _n \rangle |^2$ a node (a belly). From this, we can confirm the similarity between the nonstatic evolutions of the coherent-state wave and the Fock-state wave.

### Quantum energy and quadrature fluctuations

As the wave becomes nonstatic, the evolutions of related physical quantities may also deviate from their standard patterns. To see this in a quantitative way, lets consider quantum energy and quadrature fluctuations for instance.

As shown previously, the Hamiltonian is composed of two terms associated with electric energy ${\hat{p}^2}/{(2\epsilon )}$ and magnetic energy $\epsilon \omega ^2 \hat{q}^2/2$. From the evaluation of the expectation values of them in the coherent state using Eqs. (8) and (9), we have the formula of the electric energy and the magnetic energy from quantum-mechanical point of view as

26 $$\begin{aligned} E_\mathrm{k}= & {} -\frac{\hbar \omega }{4f(t)} \Bigg [ \left( 1+i \frac{\dot{f}(t)}{2\omega }\right) ^2 A^2 + \left( 1-i \frac{\dot{f}(t)}{2\omega }\right) ^2 A^{*2} \nonumber \\&-\left( 1+ \frac{[\dot{f}(t)]^2}{4\omega ^2}\right) (2A^*A+1) \Bigg ], \end{aligned}$$

27 $$\begin{aligned} E_\mathrm{p}= & {} \frac{1}{4} \hbar \omega f(t) [A^2 + A^{*2} + 2A^*A +1]. \end{aligned}$$

**Figure 3:**
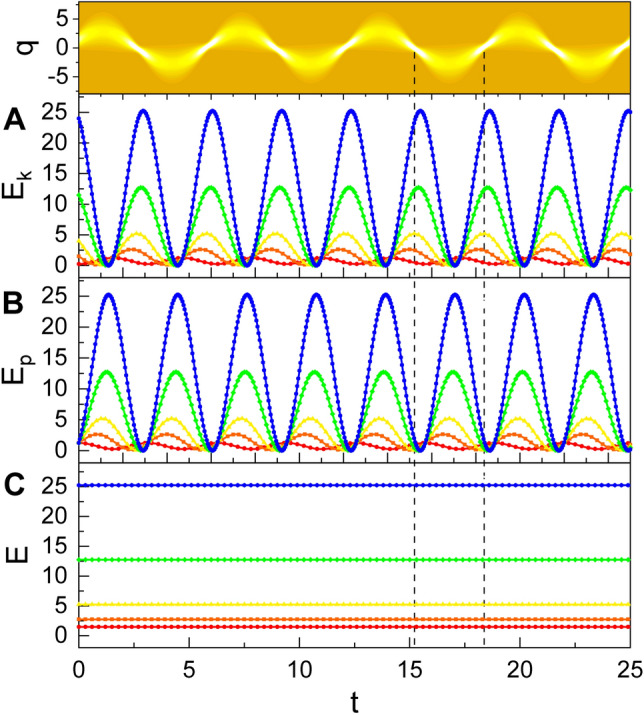
Time evolution of quantum electric energy $E_\mathrm{k}$, quantum magnetic energy $E_\mathrm{p}$, and total quantum energy *E* for several different values of $c_1$. The value of $c_1$ that we have chosen is 1 for red, 2 for orange, 4 for yellow, 10 for green, and 20 for blue lines. We have used $c_2=1$, $\omega =1$, $A_0=1$, $\hbar =1$, $\epsilon =1$, $t_0=0$, and $\varphi =\theta =0$. The evolution of the probability density for the case of $c_1=4$ is provided in upper part: this is associated to the quantities of yellow lines.

The time behaviors of these are shown in Fig. 3. Depending on the wave variation over time, both the electric and the magnetic energies vary periodically. This can be regarded as the manifestation of wave nonstaticity. The electric energy is largest at nodes, whereas the magnetic energy is largest at the bellies. However, the total quantum energy does not vary over time and this consequence agrees with the universal physical law of energy conservation.

The fluctuation of an observable $\hat{O}$ in the coherent state can be defined as $(\Delta O)_A = [\langle \hat{O}^2 \rangle - \langle \hat{O} \rangle ^2]^{1/2}$ where $\langle \cdots \rangle = \langle A|\cdots |A\rangle$. Using this, the fluctuations of canonical variables represented in Eqs. (8) and (9) are obtained, such that

28 $$\begin{aligned} (\Delta q)_A= & {} \Bigg (\frac{\hbar f(t)}{2\epsilon \omega }\Bigg )^{1/2}, \end{aligned}$$

29 $$\begin{aligned} (\Delta p)_A= & {} \Bigg [\frac{\hbar \epsilon \omega }{2 f(t)} \Bigg ( 1 + \frac{[\dot{f}(t)]^2}{4\omega ^2} \Bigg )\Bigg ]^{1/2}. \end{aligned}$$

We also readily have the corresponding uncertainty product as

30 $$\begin{aligned} (\Delta q)_A(\Delta p)_A = \frac{\hbar }{2} \Bigg ( 1 + \frac{[\dot{f}(t)]^2}{4\omega ^2} \Bigg )^{1/2}. \end{aligned}$$

**Figure 4:**
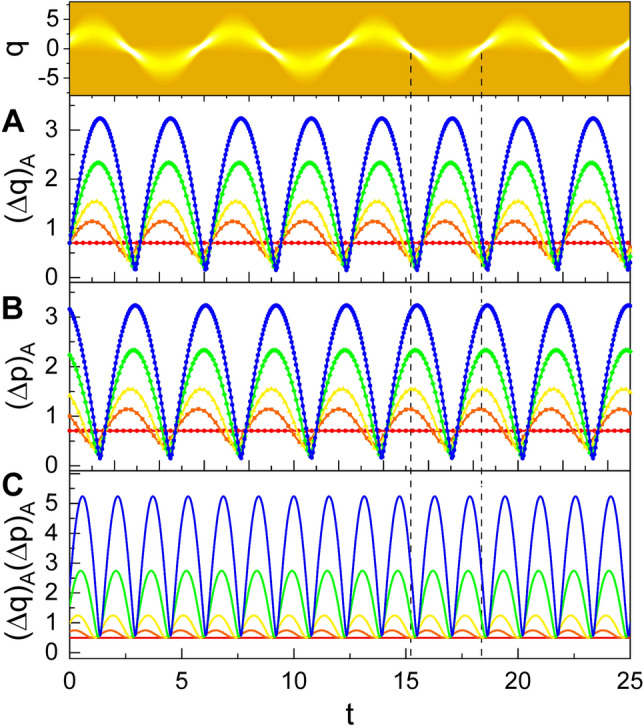
Time evolution of quadrature fluctuations $(\Delta q)_A$ (**A**), $(\Delta p)_A$ (**B**), and the uncertainty product $(\Delta q)_A(\Delta p)_A$ (**C**) for several different values of $c_1$. The value of $c_1$ that we have chosen is 1 for red, 2 for orange, 4 for yellow, 10 for green, and 20 for blue lines. We have used $c_2=1$, $\omega =1$, $\hbar =1$, $\epsilon =1$, $t_0=0$, and $\varphi =0$. In upper part, the evolution of the probability density is shown with the choice of $c_1=4$.

The time evolutions of the fluctuations $(\Delta q)_A$ and $(\Delta p)_A$ together with $(\Delta q)_A(\Delta p)_A$ are represented in Fig. 4. These also exhibit periodic behaviors over time. $(\Delta q)_A$ is largest at bellies, while $(\Delta p)_A$ is largest at nodes. On the other hand, $(\Delta q)_A(\Delta p)_A$ is smallest at both nodes and bellies. By comparing Fig. 4A with Fig. 4B, we see that the variation patterns of $(\Delta q)_A$ and $(\Delta p)_A$ are the same as each other except for the difference in the phase between them.

### Squeezing effects and nonclassicality

From Fig. 4A,B, we see that both the uncertainties $(\Delta q)_A$ and $(\Delta p)_A$ can be lowered below their standard quantum levels. If $(\Delta q)_A$ is lower than its standard quantum level, $(\Delta p)_A$ is larger than its standard quantum one and vice versa. This means that the nonstatic coherent state resembles the squeezed state on one hand. In order to see in more detail about this, let us consider the Wigner distribution function which is defined in the form

31 $$\begin{aligned} W (q,p,t) = \frac{1}{\pi \hbar } \int _{-\infty }^\infty \langle A |q+y \rangle \langle q-y |A \rangle e^{2ipy/\hbar } dy. \end{aligned}$$

A straightforward evaluation of this using Eq. (11) results in

32 $$\begin{aligned} W (q,p,t)= & {} \frac{1}{\pi \hbar } \exp \Bigg [-\zeta (t)q^2 - \Bigg ( \frac{\sqrt{\zeta (t)}}{2\omega }\dot{f}(t)q - \frac{p}{\sqrt{\zeta (t)}\hbar } \Bigg )^2 \nonumber \\&+ \sqrt{2\zeta (t)}\bigg ( (A+A^*)+i \frac{\dot{f}(t)}{2\omega }(A-A^*) \bigg )q - \frac{i}{\hbar }\sqrt{\frac{2}{\zeta (t)}}(A-A^*)p -2|A|^2\Bigg ]. \end{aligned}$$

**Figure 5:**
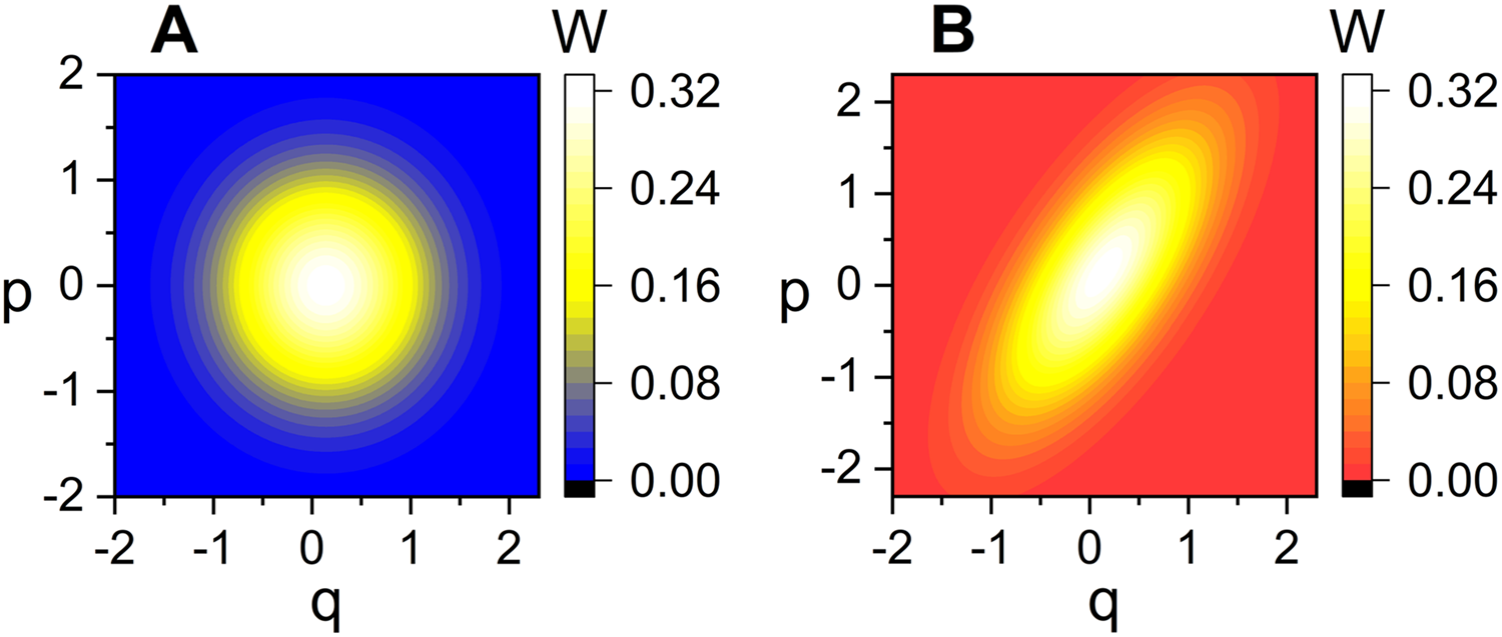
Comparison of the density plot of *W* for static (**A**) and nonstatic (**B**) light waves at $t=0$ where ($c_1$, $c_2$) are chosen as (1, 1) for A and (2, 1) for B. We have used $\omega =1$, $A_0=0.1$, $\hbar =1$, $\epsilon =1$, $t_0=0$, and $\varphi =\theta =0$.

**Figure 6:**
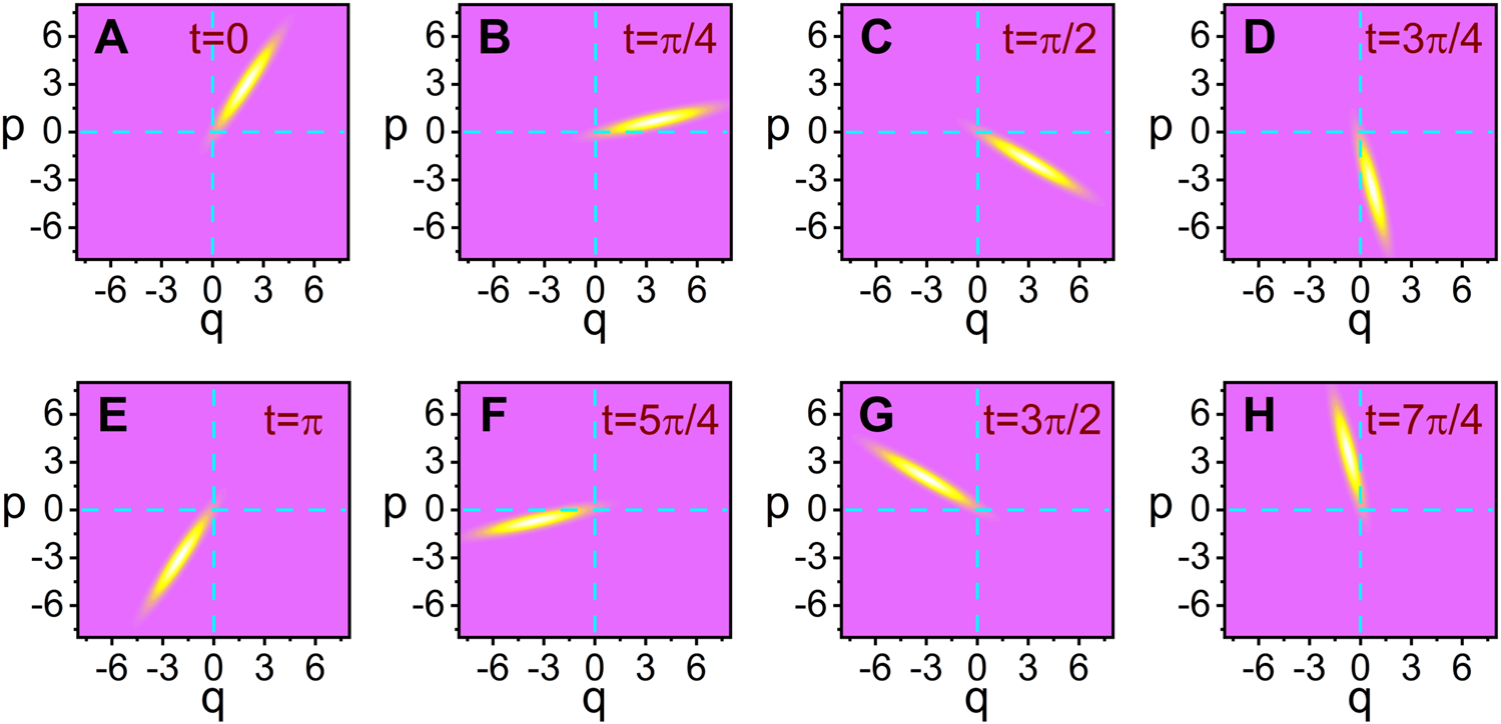
Density plots for the time evolution of *W* where the considered time is represented in each panel. The nonstaticity parameters chosen here are ($c_1$, $c_2$) = (5, 2); this choice corresponds to that of Fig. 1B. We have chosen the amplitude as $A_0=1$, which is very large compared to that in Fig. 5. All other parameters taken here are the same as those of Fig.  5.

The graphical illustration for this outcome is given in Fig. 5. By comparing Fig. 5B with Fig. 5A, we see that *W* is squeezed in a certain direction in phase space as the state becomes nonstatic. The time evolution of the Wigner distribution function for a nonstatic wave with a relatively large amplitude ($A_0$) is shown in Fig. 6. From this figure, we see that the bar that represents the squeezed distribution contour rotates as time goes by. Not only the center of the bar rotates clockwise with respect to the origin of coordinates, but the bar itself rotates clockwise about its center as well. The periods of both kinds of rotations are the same as each other and they are given by $t_\mathrm{p} = 2\pi /\omega$. Whereas the former kind of rotation is usual, the latter kind is clearly related to the fundamental light-wave nonstaticity, such as the time variation of the uncertainties and the appearance of bellies and nodes in the wave evolution. Let us look into Fig. 6 in connection with Fig. 1B that was taken the same values of $c_1$ and $c_2$. We confirm that the values of $(\Delta q)_A$ in panels B and F in Fig. 6 are relatively high, whereas they correspond to the instants of time at which the displacements in Fig. 1B is nearly highest. On the other hand, they are small in panels D and H, whereas these cases correspond to the instants where the wave forms nearly a node in Fig. 1B. This outcome agrees with the result of Fig. 4A which exhibits that $(\Delta q)_A$ is small around a node and high around a belly. From this analysis, we can understand the mechanism of squeezing that arises in the nonstatic coherent state. Squeezing effects in a quantum state is a well-known nonclassical property.

We now analyze the nonclassicality of the nonstatic state in more detail in relation to the standard description of light waves. To this end, we introduce the usual annihilation operator:

33 $$\begin{aligned} \hat{a}= \sqrt{\frac{\epsilon \omega }{2\hbar }} \hat{q} + i\frac{\hat{p}}{\sqrt{2\epsilon \omega \hbar }} , \end{aligned}$$

and its Hermitian adjoint $\hat{a}^\dagger$ that is the creation operator. Then, Eq. (6) can be rewritten in terms of them to be

34 $$\begin{aligned} \hat{A} = \mu (t) \hat{a} + \nu (t) \hat{a}^\dagger , \end{aligned}$$

where

35 $$\begin{aligned} \mu (t)= & {} \frac{1}{2\sqrt{f(t)}}\left( 1- i \frac{\dot{f}(t)}{2\omega }\right) + \frac{\sqrt{f(t)}}{2}, \end{aligned}$$

36 $$\begin{aligned} \nu (t)= & {} \frac{1}{2\sqrt{f(t)}}\left( 1- i \frac{\dot{f}(t)}{2\omega }\right) - \frac{\sqrt{f(t)}}{2}. \end{aligned}$$

Notably, $\mu (t)$ and $\nu (t)$ satisfy the relation

37 $$\begin{aligned} |\mu |^2 - |\nu |^2 =1. \end{aligned}$$

Based on the above expressions, we can also write Eqs. (28) and (29) as

38 $$\begin{aligned} (\Delta q)_A= & {} \bigg (\frac{\hbar }{2\epsilon \omega }\bigg )^{1/2} (\mu -\nu ), \end{aligned}$$

39 $$\begin{aligned} (\Delta p)_A= & {} \bigg (\frac{\hbar \epsilon \omega }{2}\bigg )^{1/2} |\mu +\nu |. \end{aligned}$$

If $\mu =1$ and $\nu =0$ (or $c_1=c_2=1$), these reduce to standard uncertainties in each quadrature, which are represented with a red curve in panels A and B in Fig. 4, respectively. Thus, we confirm that the time variation of the uncertainties shown in Fig. 4 is determined depending purely on the time variations of $\mu$ and $\nu$ that follow Eqs. (35) and (36).

**Figure 7:**
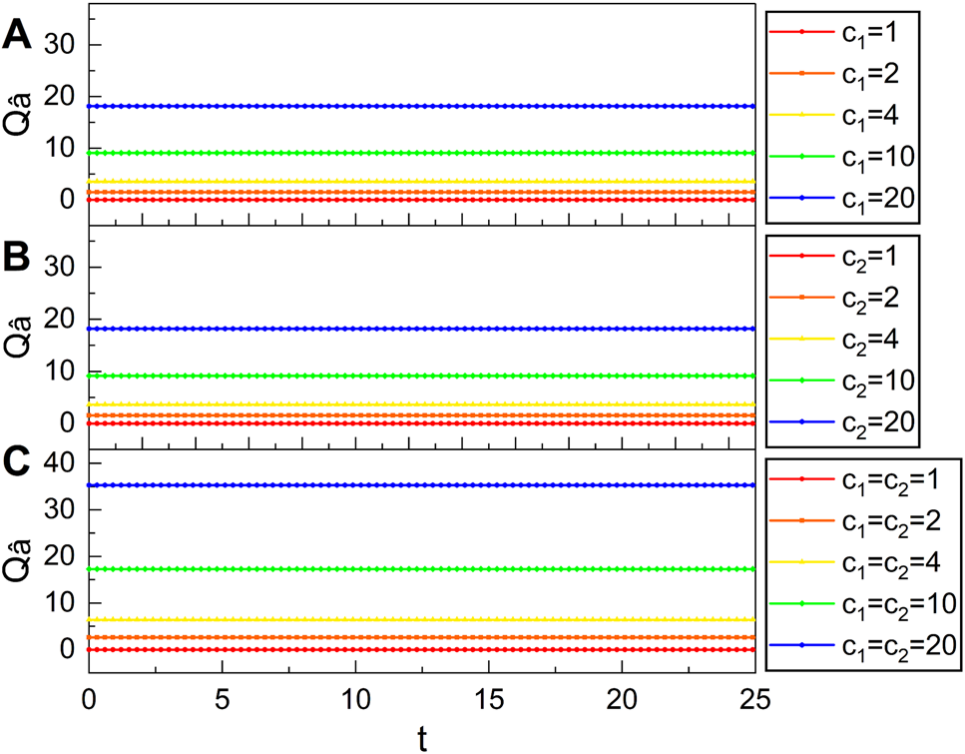
Mandel’s parameter $Q_{\hat{a}}$ versus *t* for several different values of $c_1$ (**A**), $c_2$ (**B**), and $c_1$ and $c_2$ (**C**). We have used $c_2=1$ for A, $c_1=1$ for B, $\omega =1$, $A_0=1$, $t_0=0$, and $\varphi =\theta =0$.

As a measure of nonclassicality, we see Mandel’s Q parameter^37^ for this system. $\hat{a}$ and $\hat{a}^\dagger$ can be used for estimating nonclassicality of the nonstatic state relative to the standard description of light waves because standard quantum states are described in terms of them. Considering this, it is possible to represent the Q parameter as^37^

40 $$\begin{aligned} Q_{\hat{a}} = \frac{[(\Delta (\hat{a}^\dagger \hat{a}))_A]^2 - \langle \hat{a}^\dagger \hat{a} \rangle }{\langle \hat{a}^\dagger \hat{a} \rangle }, \end{aligned}$$

where the definition of $\langle \cdots \rangle$ is still the same as the previous one which is $\langle A|\cdots |A\rangle$. While there is no upper bound for the Q parameter, its minimal value allowed in quantum mechanics is $-1$. If $-1 \le Q_{\hat{a}} < 0$, the field follows sub-Poissonian statistics, whereas it follows super-Poissonian statistics when $Q_{\hat{a}} > 0$. The light-wave description, on the other hand, reduces to Poissonian statistics in the case $Q_{\hat{a}} = 0$.

To evaluate the Q parameter, let us represent $\hat{a}$ and $\hat{a}^\dagger$ in the form

41 $$\begin{aligned} \hat{a}= & {} \mu ^* \hat{A} -\nu \hat{A}^\dagger , \end{aligned}$$

42 $$\begin{aligned} \hat{a}^\dagger= & {} \mu \hat{A}^\dagger -\nu ^* \hat{A}. \end{aligned}$$

If we insert these two formulae into Eq. (40), the problem is treated in terms of some arrays of $\hat{A}$ and $\hat{A}^\dagger$ instead of the standard ladder operators. We then rearrange the elements of Eq. (40) in a way that each array of ladder operators in their representation being the normal order^45^. Thus, with the help of Eq. (10), we eventually have

43 $$\begin{aligned} \langle \hat{a}^\dagger \hat{a}\rangle= & {} (|\mu |^2+|\nu |^2) |A|^2 -\mu \nu A^{*2}-\mu ^*\nu ^* A^{2}+|\nu |^2, \end{aligned}$$

44 $$\begin{aligned} {[(\Delta (\hat{a}^\dagger \hat{a}))_A]}^2= & {} (|\mu |^4+6|\mu |^2|\nu |^2+|\nu |^4)|A|^2 -2 (|\mu |^2+|\nu |^2)(\mu \nu A^{*2}+\mu ^*\nu ^* A^{2}) \nonumber \\&+2|\mu |^2 |\nu |^2. \end{aligned}$$

The dependence of Q parameters on $c_1$ and $c_2$ is illustrated in Fig. 7. From Fig. 7A,B, we confirm that $Q_{\hat{a}}$ for nonstatic states is larger than 0 and it increases as $c_1$ or $c_2$ grows. Figure 7C shows that the value of the Q parameter is much higher when both $c_1$ and $c_2$ are large. Interestingly, the Q parameter does not vary over time although it is represented in terms of the sinusoidal-like time function *f*(*t*). There are also many systems in which the Q parameter depends on time^38–40^. The Q parameter reduces to 0 in the standard-coherent-state limit ($c_1=c_2=1$) as expected.

Since $Q_{\hat{a}} > 0$ unless $c_1=c_2=1$, the nonstatic state considered here is described by super-Poissonian statistics. Other systems in which the photon distribution is governed by super-Poissonian statistics are found in Refs.^41–44^. While it is possible to attain photon bunching via the super-Poissonian distribution of photons, the noise in the associated photo-count is higher than the one for the standard coherent state^45^. Strong electromagnetic fields with enhanced photon bunching is important for controlling multiexciton processes in core-shell nanocrystals^41^ and intensity correlations in EIT (Electromagnetically Induced Transparency) media^42^.

## Conclusion

The quantum mechanical behavior of a nonstatic light wave in the coherent state has been analyzed. A generalized annihilation operator was introduced and its eigenfunction which plays the wave function of light was derived. We confirmed that the modification in the evolution pattern of the probability density reflects the details of the wave nonstaticity. The departure of the periodical wave evolution from that of the well-known ordinary wave becomes distinct as the degree of nonstaticity increases.

The amplitude of the wave collapses and expands in turn as a manifestation of its nonstaticity like the behavior of the Fock-state nonstatic waves. A node takes place in the graphic of wave evolution in quadrature space whenever the wave passes through $q=0$, whereas a belly takes place whenever the displacement of the wave is instantaneously largest. In fact, the instants of time where a node (or a belly) occurs are the same as those in the Fock states. The wave in conjugate *p*-quadrature space also exhibits similar pattern of nonstaticity, but the phase of its evolution precedes $\pi /2$ compared to that of the *q*-space wave.

The electric and magnetic energies of the wave also vary according to the characteristics of nonstaticity in the evolution of the wave. The electric energy is largest at the nodes, whereas the magnetic energy is largest at the bellies. However, the total wave energy does not vary over time, leading energy being conserved even if the wave exhibits nonstaticity. The fluctuations of quadratures *q* and *p* also exhibit periodic behaviors due to nonstaticity of the wave. $(\Delta q)_A$ is largest at bellies and $(\Delta p)_A$ is largest at nodes, while the corresponding uncertainty product $(\Delta q)_A (\Delta p)_A$ is in contrast smallest at both bellies and nodes.

We have confirmed that the above characteristics of wave nonstaticity in the coherent state can be explained by means of the analysis of the Wigner distribution function. As the wave becomes nonstatic, the contour of the Wigner distribution function in its phase-space plot exhibits squeezing and rotates clockwise with respect to its center. This rotation is responsible for various effects of the wave nonstaticity. We also confirmed that the Mandel’s Q parameter for the nonstatic wave is larger than unity. From this, the wave follows super-Poissonian statistics.

## Methods

### Methods summary

To describe the nonstaticity of a light wave, we introduce an invariant operator that obeys the Liouville von-Neumann equation. The invariant operator is expressed in terms of generalized annihilation and creation operators ($\hat{A}$ and $\hat{A}^\dagger$). By solving the eigenvalue equation of $\hat{A}$, we establish a coherent state that exhibits the characteristic of nonstaticity. Based on the wave function in this state, we investigate light wave nonstaticity. The quantum energy, quadrature fluctuations, the Wigner distribution function, and Mandel’s Q parameter in the nonstatic coherent state are derived using the wave function.

### Wave functions in the Fock states

Fock-state wave functions with nonstaticity are given by^21^

45 $$\begin{aligned} \langle q |\Psi _n(t) \rangle = \langle q |\Phi _n(t) \rangle \exp [i\gamma _n(t) ], \end{aligned}$$

where $\langle q |\Phi _n(t) \rangle$ are eigenfunctions of $\hat{I}$ (given in Eq. (2) or in Eq. (7)) and $\gamma _n (t)$ wave phases, of which formulae are of the form

46 $$\begin{aligned} \langle q |\Phi _n(t) \rangle= & {} \left( {\frac{\zeta (t)}{\pi }}\right) ^{1/4} \frac{1}{\sqrt{2^n n!}} H_n \left( \sqrt{\zeta (t)} q \right) \exp \left[ - \frac{1}{2}\zeta (t) \left( 1-i\frac{\dot{f}(t)}{2\omega }\right) q^2 \right] , \end{aligned}$$

47 $$\begin{aligned} \gamma _n(t)= & {} {-\omega (n+1/2) \int _{t_0}^t f^{-1} (t') dt'} + \gamma _n (t_0). \end{aligned}$$

while $H_{n}$ are *n*th order Hermite polynomials.

### The eigenfunction in *p*-quadrature space

The eigenfunction in *p*-quadrature is easily evaluated from Eq. (25) and it results in

48 $$\begin{aligned} \langle {p}|A \rangle= & {} \frac{1}{\root 4 \of {\pi \zeta }} \frac{1}{\sqrt{\hbar [1-i\dot{f}(t)/(2\omega )]}} \exp \Bigg [ -\frac{p^2+2i\sqrt{2\zeta }A\hbar p}{2\zeta \hbar ^2[1-i\dot{f}(t)/(2\omega )]} \nonumber \\&+ \frac{1+i\dot{f}(t)/(2\omega )}{2[1-i\dot{f}(t)/(2\omega )]}A^2 -\frac{1}{2}|A|^2 \Bigg ]. \end{aligned}$$
